# Cardiac Metastasis: Epidemiology, Pathophysiology, and Clinical Management

**DOI:** 10.3390/life15020291

**Published:** 2025-02-13

**Authors:** Fabiana Lucà, Iris Parrini, Maria Laura Canale, Carmelo Massimiliano Rao, Mariacarmela Nucara, Giuseppe Pelaggi, Adriano Murrone, Stefano Oliva, Irma Bisceglia, Andrea Sergi, Giovanna Geraci, Carmine Riccio, Roberto Ceravolo, Sandro Gelsomino, Furio Colivicchi, Massimo Grimaldi, Fabrizio Oliva, Michele Massimo Gulizia

**Affiliations:** 1 Cardiology Department, Grande Ospedale Metropolitano di Reggio Calabria, Bianchi Malacrino Morelli Hospital, 89129 Reggio Calabria, Italy; maricanucara@gmail.com (M.N.); giuseppepelaggi@hotmail.it (G.P.); 2 Cardiology Department, Mauriziano Hospital, 10128 Torino, Italy; irisparrini@libero.it; 3 Division of Cardiology, Azienda USL Toscana Nord-Ovest, Versilia Hospital, 55041 Lido di Camaiore, Italy; marialauracanale@icloud.com; 4 Division of Cardiology, Santa Maria Degli Ungheresi Hospital, 89024 Polistena, Italy; massimo.rao@libero.it; 5 Cardiology Unit, Città di Castello Hospital, 06012 Città di Castello, Italy; adriano.murrone@gmail.com; 6 Cardiology Unit, IRCCS Istituto Tumori “Giovanni Paolo II”, 70124 Bari, Italy; stefanoliva66@gmail.com; 7 Integrated Cardiology Services, Department of Cardio-Thoracic-Vascular, Azienda Ospedaliera San Camillo Forlanini, 00152 Rome, Italy; irmabisceglia@gmail.com; 8 Radiology Unity, Grande Ospedale Metropolitano di Reggio Calabria, 89129 Reggio Calabria, Italy; andreasergi82@icloud.com; 9 Cardiology Department, Sant’Antonio Abate Hospital, ASP Trapani, 91100 Erice, Italy; giovannageraci@hotmail.com; 10 Cardiovascular Department, Sant’Anna e San Sebastiano Hospital, 95122 Caserta, Italy; carmine.riccio8@icloud.com; 11 Cardiology Unit, Giovanni Paolo II Hospital, 88046 Lamezia, Italy; roberto_ceravolo@yahoo.it; 12 Cardiovascular Research Institute, Maastricht University, 6211 LK Maastricht, The Netherlands; sandro.gelsomino@gmail.com; 13 Clinical and Rehabilitation Cardiology Department, San Filippo Neri Hospital, ASL Roma 1, 00193 Roma, Italy; furio.colivicchi@gmail.com; 14 Cardiology Department, F. Miulli Hospital, Acquaviva delle Fonti, 70021 Bari, Italy; m.grimaldi@miulli.it; 15 Cardiology Unit, ASST Grande Ospedale Metropolitano Niguarda, 20162 Milano, Italy; fabri.oliva@gmail.com; 16 Cardiology Department, Garibaldi Nesima Hospital, 95122 Catania, Italy; michele.gulizia60@gmail.com

**Keywords:** cardiac tumors, metastatic tumors, heart, diagnostic method

## Abstract

Cardiac metastases (CMs) are more common than primary cardiac tumors, with autopsy studies reporting incidence rates between 2.3% and 18.3%. Their increasing detection is largely attributed to advances in cancer treatments, which have extended patient survival. CMs may present with diverse clinical manifestations depending on their size, location, and extent of infiltration, although they often remain asymptomatic and are identified only postmortem. Sometimes, they are incidentally discovered during tumor staging or follow-up evaluations. This review explores the incidence, pathophysiology, clinical features, and potential complications of CMs, focusing on their prevalence and characteristics. It highlights the importance of early detection and optimized management strategies to address this growing clinical concern. Further research is essential to elucidate the mechanisms driving CMs and develop effective therapeutic interventions.

## 1. Introduction

Cardiac metastases (CMs) are more prevalent than primary cardiac tumors (PCTs), with a frequency reported to be 20 to 40 times higher. Autopsy findings indicate an incidence ranging from 2.3% to 18.3%, with up to 9.1% of cases occurring during active disease [1]. Furthermore, a notable proportion (14.2%) of patients with widespread metastatic disease exhibit cardiac involvement [2]. Virtually all tumor types possess the potential to metastasize to the heart. However, cardiac involvement is influenced by several factors, primarily the tumor type, aggressiveness, and capacity for cardiac dissemination. In adults, certain non-cardiac primary malignancies, such as mesothelioma and melanoma, are well-known for their propensity to metastasize to the heart. Additional malignancies commonly associated with cardiac metastasis include lung, breast, and esophageal cancers [1], with melanoma demonstrating the highest tendency for cardiac involvement. In pediatric populations, the most common sources of CMs are lymphoma, leukemia, Wilms’ tumor, neuroblastoma, hepatoblastoma, and sarcoma [3,4].

Malignant tumors reach the heart through one of four primary routes: hematogenous spread, lymphatic dissemination, transvenous extension, or direct invasion from adjacent structures such as the lungs or mediastinum [5]. The metastatic route plays a critical role in determining the specific cardiac tissues affected [6]. For instance, tumors spreading via the lymphatic system frequently involve the pericardium or epicardium [7]. Among the cardiac structures, the pericardium is the most affected site, while the involvement of the myocardium, endocardium, large vessels, or coronary arteries is rare [7].

Pericardial involvement in CMs may present as pericardial effusion, which can progress to life-threatening cardiac tamponade [8]. Myocardial metastases, on the other hand, can lead to conduction disturbances, resulting in arrhythmias that may be refractory to standard antiarrhythmic therapies [9]. Additionally, myocardial infiltration by neoplastic cells can eventually lead to heart failure. Intracavitary masses may obstruct blood flow, causing valvular dysfunction [10]. While multiple masses or nodules are typical of metastatic disease, diffuse infiltration has also been reported [11].

Autopsy studies suggest that up to 12% of oncology patients have metastases involving the heart or pericardium, though most of these cases remain clinically silent [7]. Consequently, cardiac involvement should be considered—and actively investigated—in any patient with a known malignancy who develops new cardiovascular signs or symptoms [12]. However, these lesions often remain underdiagnosed for extended periods.

The differential diagnosis of PCTs includes both benign and malignant lesions. When intracardiac lesions are detected on ultrasound, differential considerations should include thrombi, vegetations, and foreign bodies. Myocardial metastases should be considered in the differential diagnosis of acute myocardial infarction (AMI), while pericardial metastases should be part of the differential diagnosis for pericardial effusion [13]. Early diagnosis and improved management through a multidisciplinary approach can contribute to better survival outcomes for these patients [14].

The objective of this review is to provide a comprehensive overview of secondary cardiac involvement, spanning from epidemiology to clinical management, with the aim of serving as a practical guide for cardiologists and clinicians engaged in the multidisciplinary care of cancer patients.

## 2. Materials and Methods

### 2.1. Search Strategy

The literature review was conducted in accordance with the guidelines established by the Preferred Reporting Items for Systematic Reviews and Meta-Analyses (PRISMA) Guidelines [15] and the Cochrane Handbook [16]. This paper has been registered on Prospero, with the number CRD42025638301.

A comprehensive search of the literature was performed across PubMed, Web of Science, and Google Scholar, without any restrictions. PubMed was designated as the primary database. The search terms used in PubMed were: (“cardiac metastasis” [Mesh] OR “Cardiac Metastasis”) AND (“heart metastasis” [Mesh] OR “Heart Metastasis”). Only English-language articles were included in the review. The search strategy was developed by two authors (F.L. and I.P.) and subsequently approved by a third author (M.L.C.). The literature search itself was carried out by one author (C.M.R.), while two independent reviewers (G.P. and M.C.N.) assessed the eligibility of the articles and evaluated the risk of bias. The risk of bias at the individual study level was assessed using the Risk of Bias in Non-Randomized Studies of Interventions (ROBINS-I) tool [17]. Two reviewers (A.M. and C.R.) independently evaluated the included studies for risk of bias. In cases of disagreement, a third reviewer (G.G.) was consulted to achieve consensus. Bias was assessed according to the following criteria: (1) confounding factors, (2) selection of participants, (3) classification of interventions, (4) deviations from intended interventions, (5) missing data, (6) outcome measurement, (7) selection of reported outcomes, and (8) overall bias. The evaluation was conducted in line with the guidelines set forth in the Cochrane Handbook. Risk-of-Bias VISualization (robvis) software—v0. 3.0 was used to create the ROBINS-I bias assessment plot [18].

### 2.2. Selection Process

The selection of articles was guided by specific inclusion criteria, which were as follows: (1) studies involving human subjects; (2) full-text articles focusing on [topic] with a non-control population; (3) studies providing sufficient information about the presence of cardiac metastasis; and (4) studies including a minimum of 10 patients.

The exclusion criteria were the following: (1) non-human studies, (2) case reports, (3) previous reviews and/or meta-analyses, (4) editorials, (5) studies lacking [specific data], (6) studies reporting [specific outcome], and (7) studies without data related to [specific aspect].

## 3. Results

**Figure 1:**
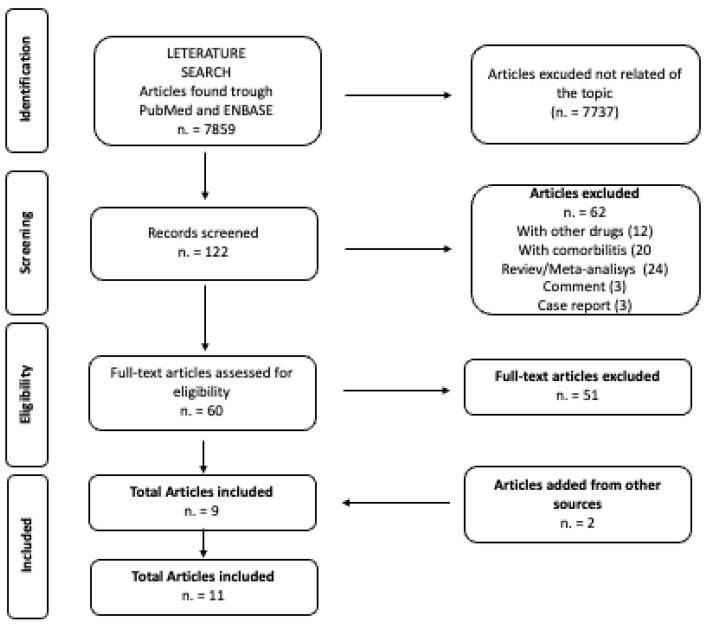
PRISMA diagram on the selection of included studies.

The initial search resulted in 2676 articles published over five years. After applying the inclusion and exclusion criteria, 2355 articles were excluded due to irrelevance, leaving 321 articles for screening based on their titles and abstracts. Following this initial screening, 253 articles were selected for further evaluation. Ultimately, six articles met our eligibility criteria (see flowchart Figure 1). Therefore, six studies were included in the final analysis, and a summary of these studies is presented in Table 1.

The PRISMA flow diagram is shown in Figure 1.

## 4. Incidence

CMs can arise from any malignant tumor capable of spreading to distant sites. While CMs are relatively rare, their incidence rate is higher than those of primary cardiac tumors, which have been estimated to range from 0.2% to 1.7%. Indeed, the incidence of CMs has been reported to range from 2.3% to 18.3% [18,19,20,21,22,23,24,25,26,27,28,29,30,31,32,33] (see Table 2). While any malignant tumor has the potential to metastasize to the heart, certain tumors, such as melanoma and primary tumors of the mediastinum, have a greater propensity to do so. Nonetheless, the ability of a tumor to metastasize to the heart is influenced by multiple factors; these include the characteristics of the primary tumor, as well as the specific histological and functional properties of the heart.

The most frequently reported origin of CMs is lung carcinoma, followed by malignancies of the blood and breast.

CMs are typically classified into three categories: pericardial, epicardial–myocardial, and intraluminal. Of these, pericardial involvement is the most observed, generally resulting from the retrograde spread of malignant cells via mediastinal lymphatic channels. Myocardial metastases usually occur through hematogenous dissemination and are most frequently associated with melanoma and hematologic cancers. In contrast, intraluminal metastases, which are characterized by the extension of tumor cells through the venous system into the heart chambers, are most commonly seen in cases of renal cell carcinoma, adrenocortical carcinoma, and hepatocellular carcinoma.

## 5. Pathophysiology

The pathophysiology underlying CMs encompasses several mechanisms. The spread of a primary tumor can potentially affect any structure of the heart, including the coronary arteries, pericardium, endocardium, great vessels, and myocardium. Four main pathways of cardiac involvement are recognized in this context. The first pathway is a direct extension from primary malignancies. This occurs through direct contact with adjacent organs such as the lungs, pleura, breast, and esophagus. In these cases, the pericardium is typically the most affected due to direct invasion by an intrathoracic or mediastinal tumor. Importantly, it should be highlighted that the local invasiveness of a tumor should not be confused with metastatic dissemination. Indeed, when a tumor grows and extends into surrounding tissues, it demonstrates local invasiveness, which differs from metastatic dissemination. In metastatic dissemination, cancer cells detach from the primary tumor, migrate through the bloodstream or lymphatic system, and establish secondary tumors in distant organs. While both phenomena involve the spread of cancer, local invasion remains restricted to adjacent structures, whereas metastasis involves the formation of new tumors at distant sites. In the case of CMs, when a direct infiltration from nearby structures (such as the lungs) occurs, the heart or major vessels (e.g., superior vena cava, pulmonary veins) may be invaded from adjacent organs, leading to superior vena cava syndrome or venous obstruction.

The second pathway involves hematogenous dissemination, which is characteristic of malignancies like melanoma, lymphoma, and sarcoma [34]. In this scenario, metastatic cells circulating in the bloodstream can lodge within the cardiac chambers, often leading to endocardial metastases [35].

The third and most common pathway for metastatic cells to reach cardiac structures is through the lymphatic system. The relatively lower incidence of secondary tumors in the heart, compared with other organs, can be attributed to the complex network of cardiac lymphatics, which includes subepicardial, myocardial, and subendocardial networks. The flow of lymph within the heart moves from the endocardium towards the epicardium, with a web of capillaries covering the entire heart surface. Perpendicular to this network, lymphatic capillaries traverse the myocardium parallel to the vascular supply.

A unique aspect of the cardiac lymphatic system is that the flow within these capillaries and ducts is influenced by the pressure changes associated with cardiac diastole and systole. In the presence of CMs, neoplastic emboli can obstruct intramural lymphatics, leading to lymph stagnation in the myocardial regions upstream of the obstruction. This obstruction can impair endocardial-to-epicardial lymphatic drainage, resulting in tissue damage due to lymph stasis and edema, which promotes increased proliferation of neoplastic cells in undrained regions. Additionally, retrograde lymph flow may disseminate metastases to deeper cardiac structures. The increased pressure may also cause the lymphatic walls to rupture, leading to interstitial tumor spread.

Once the lymphatics are invaded by neoplastic cells, myocardial contractions have a dual effect on forming cardiac metastases. On the one hand, contractility hinders the spread of intramural tumor metastases by facilitating lymph and blood drainage, thereby displacing cardiac tumor-produced emboli. On the other hand, it aids the diffusion of neoplastic cells along the epicardial surface. Given that the lymphatic system represents the primary route for cardiac metastases, the obstruction of common lymphatic nodes by neoplastic cells from metastasized mediastinal lymph nodes is critical in forming cardiac metastases.

**Figure 2:**
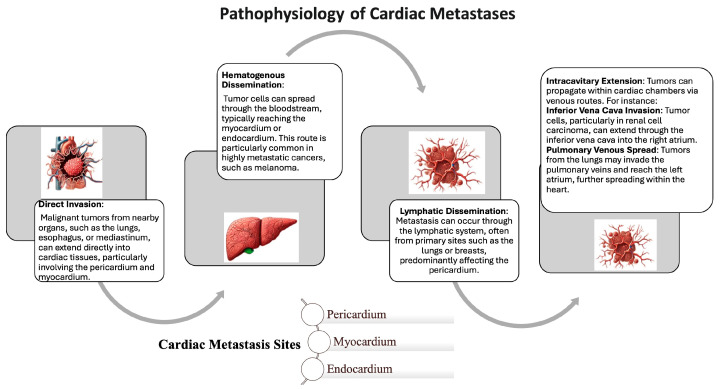
**Pathophysiology of Cardiac Metastases**. Metastasis to the heart occurs when tumor cells from a distant organ spread through hematogenous, venous, or lymphatic routes, or by direct extension. In lymphatic spread, cells travel via lymphatic vessels and reach the heart through lymphatic drainage.

Following the colonization of the epicardium by neoplastic cells, emboli can penetrate the intramural lymphatics. Penetration into the myocardium is facilitated by lymphatic stasis resulting from epicardial involvement, damage to the epicardial plexus due to stasis, and the direct effects of neoplastic cells. In many cases, the toxic effects of chemotherapy also contribute to this process. Figure 2 illustrates the potential pathophysiological mechanisms underlying cardiac metastasis and subsequent cardiac dysfunction.

Lymphatic spread from lung and breast malignancies also contributes to the development of CMs [36]. In addition, intracavitary spread through the inferior vena cava or pulmonary veins is implicated in CMs originating from renal and hepatocellular carcinoma [37]. Notably, renal cell carcinoma and lung cancer may, respectively, infiltrate the right and left atria via the inferior vena cava and pulmonary veins [38].

## 6. Endocardial, Myocardial, and Epicardial Metastasis

Endocardial metastases are rare and typically arise from either the direct invasion of the pericardium or hematogenous dissemination. These metastases predominantly affect the right cardiac chambers due to their lower flow and contraction compared to the left chambers. They often originate from the inferior vena cava, associated with tumors of the kidney, liver, and uterus, or from the superior vena cava, linked to thyroid tumors, primarily involving the right atrium. Clinically, they may present as elongated, highly mobile masses that resemble tumor thrombi. Contrast echocardiography can be helpful in identifying such masses, as the ventricle appears opaque. In contrast, the mass initially remains dark, gradually becoming more visible as contrast is absorbed by the tumor. Transesophageal echocardiography (TEE) and three-dimensional echocardiography (3DE) provide enhanced imaging clarity, especially for atrial masses [39,40].

**Figure 3:**
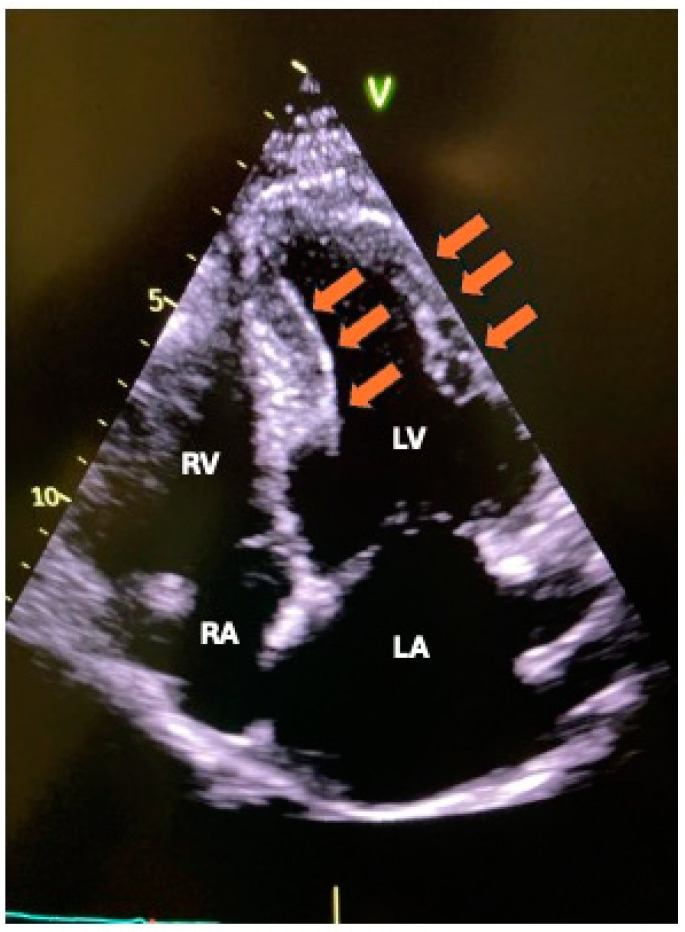
Lymphoma with direct myocardial invasion: four-chamber view showing lymphoma invading the entire left ventricular wall (arrows). RV: right ventricle; RA: right atrium; LV: left ventricle; LA: left atrium.

Myocardial metastases occur in 29% to 32% of cases and typically result from direct pericardial extension, lymphatic dissemination, or hematogenous spread. When the myocardium is involved, metastases often remain asymptomatic or present with atypical symptoms. The symptomatology usually correlates with the extent of infiltration, leading to segmental myocardial alterations that may precipitate heart failure (HF) or trigger atrial or ventricular arrhythmias or conduction disturbances [7]. Both lymphoma and sarcoma tend to infiltrate significant areas of the myocardium, often remaining asymptomatic despite substantial loss of contractile tissue [7]. (Figure 3). However, clinical manifestations can advance to HF [7] in cases of extensive ventricular infiltration.

Additionally, the formation of neoplastic emboli can lead to embolization within the coronary arteries, potentially resulting in acute myocardial infarction (AMI). Metastases to the myocardium generally exhibit echogenicity that differs from that of the surrounding myocardial tissue [7]. The injection of a contrast medium, particularly when myocardial echogenicity appears irregularly enhanced, may aid in visualization [7]. Metastasis to the epicardium is observed in approximately 25% to 34% of cases, often resulting from lymphatic or hematogenous dissemination from the myocardium [35].

## 7. Clinical Manifestations

The clinical manifestations of CMs vary according to their location, size, and extent of infiltration. Often, these metastases remain asymptomatic and are only identified postmortem; however, they may also be discovered incidentally during tumor staging procedures1. Common symptoms associated with CMs include dyspnea, tachycardia, chest pain, and syncope. Sudden death can occur due to acute myocardial infarction (AMI), ventricular wall rupture, or arrhythmias [1,2,3,4,5,6,7,8,9,10,11,12,13,14,15,16,17,18,19,20,21,22,23,24,25,26,27,28,29,30,31,32,33,34,35,36,37,38,39,40,41].

It is important to note that dyspnea, tachypnea, and tachycardia may be complicated by concomitant anemia and hypoproteinemia. Additionally, since thoracic cancers—particularly lung cancer—can present with similar symptoms, recognizing cardiac involvement in these conditions may be challenging. Symptoms suggestive of potential cardiac tamponade include hypotension, dyspnea, paradoxical pulse, Kussmaul’s sign, and jugular distension [42].

Metastatic growth within the myocardium can lead to arrhythmias and conduction blockages, as well as left ventricular dysfunction. Moreover, embolization or invasion of the coronary arteries may result in angina or acute myocardial infarction [9]. Systemic embolisms affecting the pulmonary, peripheral, or cerebral circulation may induce additional symptoms.

Physical examination findings associated with CMs may include pericardial rubs, muffled heart sounds, elevated jugular venous distention, hepatomegaly, edema, and pulsus paradoxus. While electrocardiogram (ECG) findings typically lack specificity, persistent ST-segment elevation without Q waves may indicate myocardial infiltration [9].

## 8. Challenges in Diagnosing Cardiac Metastases in Asymptomatic Patients

Diagnosing CMs in asymptomatic patients is particularly challenging due to their infrequent occurrence and the subtlety of their presentation. Indeed, symptoms are often not specific and mimic primary cardiac diseases, complicating a correct diagnosis [1]. Therefore, these lesions are often uncovered incidentally during imaging for unrelated clinical evaluations, as symptoms generally emerge only in advanced stages. Radiologists should maintain a high level of awareness when interpreting imaging studies, as subtle anatomical changes or atypical contrast enhancement may indicate cardiac involvement. Imaging techniques, including echocardiography, cardiac magnetic resonance imaging (CMRI), and computed tomography (CT), are essential tools, though their utility often depends on clinical suspicion [12]. Positron emission tomography (PET) imaging with 2-deoxy-2-[fluorine-18]fluoro-D-glucose (18F-FDG) can also assist in identifying cardiac involvement, although uptake variability within the myocardium—affected by factors such as fasting state and glucose levels—may be an obstacle to detection. Careful analysis of abnormal cardiac uptake on FDG-PET can improve early identification of metastatic lesions [1]. Early detection is crucial to integrate CMs into treatment planning and reduce the risk of severe cardiac complications.

## 9. Pericardial Involvement

Pericardial effusion is frequently observed in cancer patients but is not always directly linked to pericardial involvement by the malignancy. In fact, up to 30% of pericardial effusions may result from chemotherapy or radiotherapy side effects, idiopathic pericarditis, or opportunistic infections. Pericardial involvement in cardiac CMs can range from minimal infiltration to extensive dissemination, with the volume of effusion varying from small amounts to large collections that may lead to cardiac tamponade [43].

Pericardial involvement typically occurs through direct invasion by thoracic cancers, but can also result from mediastinal lymphatic obstruction or treatment-related complications [43]. A study by Butany, which reviewed 11,432 autopsies and included 266 cases of cardiac neoplasms, found that the pericardium was involved in 64% to 69% of metastatic cases. Pericardial effusion may be the first sign of cardiac tumors, often presenting as moderate to severe and frequently hemorrhagic due to capillary damage caused by tumor-related substances. When a neoplastic cause is suspected, pericardiocentesis is recommended. The rapid accumulation of hemorrhagic fluid after drainage strongly suggests a malignant etiology [43].

For diagnostic accuracy, pericardial fluid should be promptly analyzed by a pathologist, with immediate centrifugation as the preferred method. If centrifugation is not immediately available, the fluid should be refrigerated at 4 °C. The gold standard for diagnosing malignant pericarditis is detecting neoplastic cells in the fluid, with a minimum of 60 mL often required for cytological examination [44]. The sensitivity of cytology ranges from 71% to 92%, with a specificity of 100%.

Tumor markers in the pericardial fluid can also aid diagnosis, with results available within hours. Claudin-4 detection is beneficial for distinguishing mesothelioma from carcinoma in patients with known malignancy. Commonly tested markers include carcinoembryonic antigen (CEA), cytokeratin fragment 19 (CYFRA 21-1), and neuron-specific enolase (NSE). Elevated levels of CEA (>10 ng/mL) and CYFRA 21-1 (>100 ng/mL) strongly indicate neoplastic pericarditis. Notably, high CYFRA 21-1 and low CEA levels in pleural fluid may suggest mesothelioma [45]. Other specific markers, such as CA 19-9, CA 125, CA 15-3, BRST-2, and p63, are available but have shown limited sensitivity for diagnosis. A pericardial biopsy may be warranted if cytological results are negative, though its sensitivity is often lower [46].

**Figure 4:**
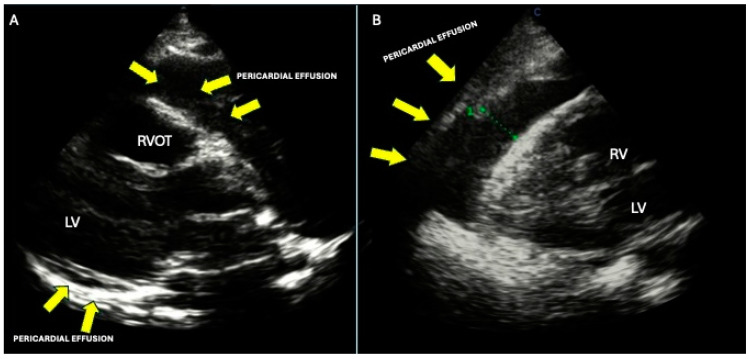
(**A**,**B**) A 24-year-old patient diagnosed with Hodgkin lymphoma presented with clinical signs of cardiac tamponade. (**A**) The image illustrates a parasternal long-axis echocardiographic view, showing a circumferential pericardial effusion with significant accumulation, predominantly along the right ventricular free wall and the posterior wall of the left ventricle. (**B**) The figure demonstrates a subxiphoid echocardiographic view, highlighting a pericardial effusion exerting compressive effects on the right ventricle, indicative of hemodynamic compromise. RVOT: right ventricular outflow tract; LV: left ventricle; RV: right ventricle.

Electrocardiographic (ECG) findings in pericarditis are typically non-specific, and pericardial effusion may present with a low QRS voltage and electrical alternans. Echocardiography is the primary imaging modality for diagnosing pericardial effusion and assessing its hemodynamic impact. Criteria suggestive of malignancy include infiltration, tumor mass location (particularly if not on the left side), and significant effusion. Pericardial effusions are classified as mild (<10 mm), moderate (10–20 mm), or large (>20 mm). Signs of cardiac tamponade include a “swinging” heart, compression of the right-sided heart chambers, abnormal interventricular septal movement, respiratory variation in mitral E velocities (>25%) and tricuspid E velocities (>40%), and inferior vena cava distension [47] (Figure 4A,B). Recurrent pericarditis can lead to pericardial thickening and progress to constrictive pericarditis. Additionally, echocardiography may detect intrapericardial masses.

The clinical presentation of pericardial effusion is crucial in determining therapeutic strategies, particularly when cardiac tamponade is suspected [39]. Pericardiocentesis, either via percutaneous or surgical drainage, is often required, with echocardiography guiding the approach (subcostal, apical, or parasternal) based on the fluid’s location. Management of pericardial metastases and preventing effusion recurrence typically involve a multidisciplinary approach tailored to individual patient needs and disease characteristics.

In cases where echocardiographic assessment is insufficient, computed tomography (CT) can guide pericardiocentesis [39]. Surgical drainage may be necessary when percutaneous techniques are inadequate. Recurrence prevention strategies include prolonged pericardial drainage, pericardial sclerosis, and the creation of pleuropericardial or pleuroperitoneal windows through percutaneous balloon pericardiotomy or surgery. Systemic or intrapericardial chemotherapy or radiotherapy may also help reduce the recurrence risk. Prolonged drainage can be maintained by connecting the catheter to a sterile bag, although this increases the risk of catheter displacement or blockage.

Historically, tetracycline instillation into the pericardial space was used for sclerosis, but it carried higher risks of side effects, including fever, pain, and atrial arrhythmias [48]. Recently, bleomycin and thiotepa, both sclerosing antineoplastic agents, have gained favor, while cisplatin has been effective for pericardial effusion associated with lung cancer [1,2,3,4,5,6,7,8,9,10,11,12,13,14,15,16,17,18,19,20,21,22,23,24,25,26,27,28,29,30,31,32,33,34,35,36,37,38,39,40,41,42,43,44,45,46,47,48,49].

The pleuropericardial window, which creates a direct communication between the pericardium and pleura to drain pericardial fluid, can be performed surgically or percutaneously using a balloon catheter [50]. Studies suggest this approach provides good outcomes, though it carries a higher complication risk than pericardial drainage alone, including adhesive pericarditis and loculated effusions requiring further surgical intervention.

Intrapericardial chemotherapy, either alone or in combination with systemic chemotherapy, has demonstrated efficacy, particularly in cancers that spread via the retrograde lymphatic pathway, such as lung and breast cancers [1].

## 10. Diagnostic Imaging

**Figure 5:**
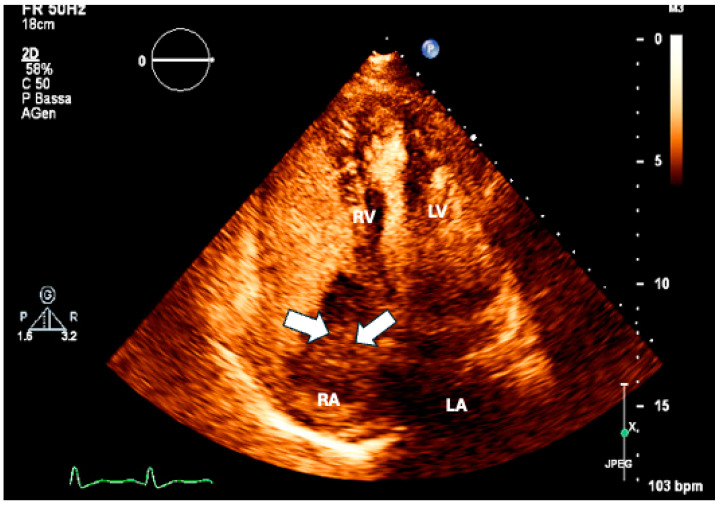
A contrast-enhanced view of lymphoma metastases: a four-chamber view showing metastasis in the right atrium. The two arrows indicate metastatic masses within the right atrium, which appear as echogenic areas due to contrast enhancement, distinguishing them from potential vascular thrombi. RV: right ventricle; RA: right atrium; LV: left ventricle; LA: left atrium.

Transthoracic echocardiography (TTE) plays a crucial role in diagnosing and differentiating various characteristics of cardiac masses. It enables localization, size determination, infiltration assessment, and mobility evaluation [51]. Furthermore, echocardiography aids in distinguishing between metastases and thrombi [51]. Metastatic masses typically exhibit contrast enhancement, distinguishing them from other structures like vascular thrombi [52] (Figure 5). In Figure 5, the metastatic invasion highlighted by the arrows appears as a well-defined, bright echogenic area due to its ability to retain the contrast agent more effectively than surrounding normal cardiac tissue. This is a key diagnostic feature, as vascular thrombi, in contrast, generally do not enhance with contrast agents and often appear as hypoechoic (darker) or poorly defined structures within the chamber.

This differentiation is critical in clinical practice for accurate diagnosis and guiding subsequent therapeutic strategies.

Transesophageal echocardiography (TEE) offers superior visualization of the cardiac chambers compared with TTE [53]. It enables the detection of small masses (<5 mm) that may not be adequately visualized using 2D TTE. Additionally, TEE provides enhanced visualization of cardiac structures, particularly in cases of suspected masses or lesions [53].

Three-dimensional echocardiography (3D echo) provides precise visualizations of lesions and their involvement with adjacent cardiac structures [54]. It offers comprehensive imaging, enabling the accurate assessment of tumor morphology and spatial relationships with surrounding tissues [54].

**Figure 6:**
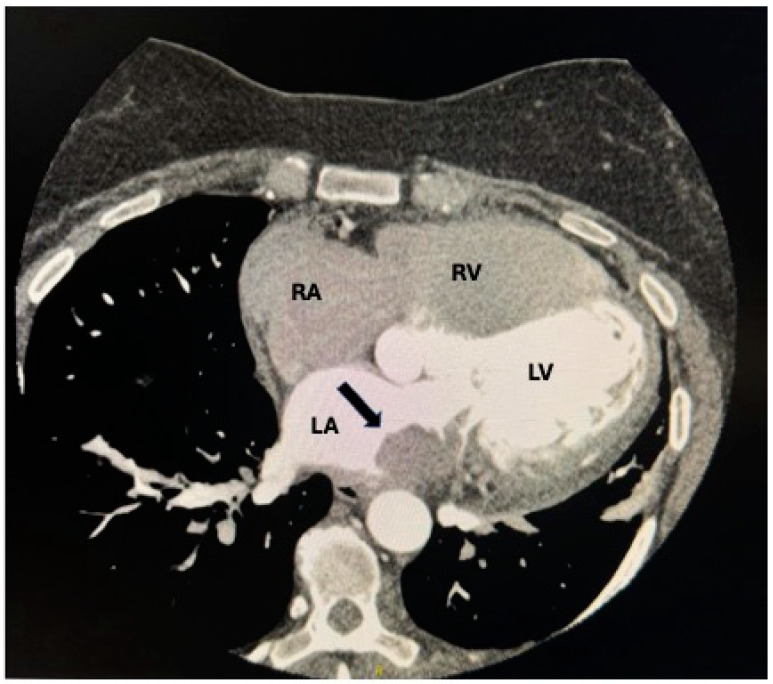
A contrast-enhanced CT image showing an endoluminal mass in the left atrium, likely metastatic from renal carcinoma. The black arrow indicates pathological tissue within the left atrium during the arterial phase following iodinated contrast infusion. RA: right atrium; LA: left atrium; LV: left ventricle; RV: right ventricle.

Computed tomography (CT) scanning is valuable for delineating the morphology of cardiac tumors and their relationship with neighboring structures [39]. It provides detailed anatomical information, which aids both the diagnosis of cardiac masses and the planning of any surgical treatment [39] (Figure 6).

Cardiac magnetic resonance imaging (CMRI) offers tissue characterization capabilities which facilitate the differentiation between infiltrating metastases and normal myocardium. It provides information regarding tumor size, morphology, localization, and relationships with adjacent tissues [55]. Metastases typically exhibit a low T1 signal and an enhanced T2-weighted signal on CMRI. Furthermore, CMRI can visualize the direct extension of tumors from the mediastinum and identify surrounding structures [55].

Positron emission tomography (PET) relies on fluorodeoxyglucose (FDG-PET) to visualize metabolic activity and is commonly utilized in detecting metastatic tumors. However, PET imaging may pose challenges due to the high metabolic activity of the myocardium, because this may mimic tumor activity [1,56].

Endomyocardial biopsy can be considered in the absence of a primary tumor diagnosis and may aid in treatment planning, particularly for chemotherapy [57].

During differential diagnosis, it is crucial to consider other conditions such as thrombus, pericardial cyst, infection, or adverse effects of chemotherapy or radiotherapy in cases of pericardial effusion (Table 3).

## 11. Management

Treatment strategies for CMs depend on the histology of the primary tumor and the extent of cardiac involvement [58]. Systemic chemotherapy is the preferred modality for lymphoma, while local radiotherapy remains a viable alternative. Surgical intervention is reserved for specific cases where metastases cause significant cardiac functional impairments or when the tumor’s origin remains unclear.

However, the management of CMs remains a therapeutic challenge due to a lack of established guidelines, necessitating input from a multidisciplinary team. Surgery is typically considered for patients with ventricular impairment and a relatively favorable prognosis who are in good general health. Surgical resection is generally indicated when the complete removal of the tumor is technically feasible or in cases of intracardiac obstruction [18,19,20,21,22,23,24,25,26,27,28,29,30,31,32,33,34,35,36,37,38,39,40,41,42,43,44,45,46,47,48,49,50,51,52,53,54,55,56,57,58,59]. To perform heart surgery can be complex, as it must account for potential post-surgical complications. Intracardiac obstruction caused by CMs may require surgical intervention, although outcomes are often poor if ventricular function has been irreversibly compromised. Nonetheless, the resection of CMs theoretically provides maximum local control [60].

In addition to surgery, radiotherapy and chemotherapy are both helpful in treating CMs. However, data on palliative radiotherapy are limited. The use of palliative radiotherapy in these patients is often constrained by technical challenges that limit the safe delivery of clinically effective radiation doses to the heart, as well as concerns regarding radiation-induced toxicity [41]. Risks associated with radiotherapy include the radiation dose, the duration of treatment, and the radiation volume. Re-irradiation for CMs is particularly challenging due to the potential for late cardiotoxicity, especially in patients previously treated with anthracyclines, 2D radiotherapy, or 3D conformal radiotherapy for sternal bone metastases.

Given the long-term hematological and cardiac toxicities associated with anthracyclines, the selection of chemotherapy regimens and dose density in breast cancer patients must consider several tissue biomarkers, which can be assessed through immunohistochemistry (IHC) techniques to enable personalized treatment [61,62,63,64]. Although cardiac radiotherapy for radiosensitive cardiac tumors appears to be a reasonable treatment option, its use in clinical practice is rare. This is likely due to the infrequency of clinically evident cardiac tumors as well as concerns over radiation-induced cardiac toxicity [19]. Despite its inability to cure metastatic cancer, cardiac radiotherapy can provide transient local tumor control and symptomatic relief and may improve quality of life.

**Figure 7:**
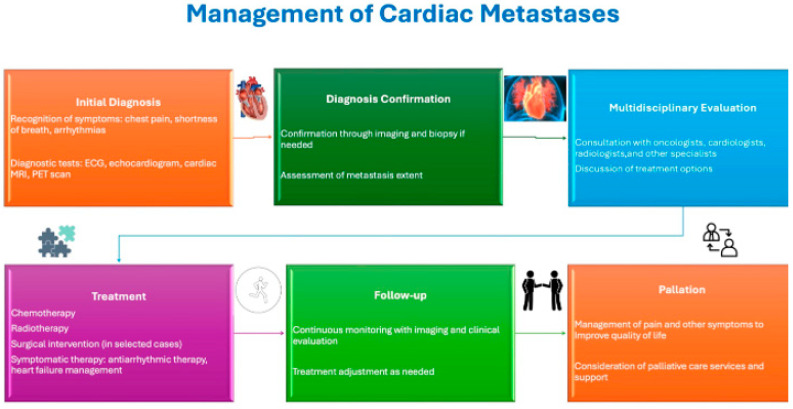
Clinical management of CMs should be individualized, focusing primarily on treating the primary tumor. Surgery is usually not recommended, except in cases of intracavitary metastases causing severe hemodynamic issues or cardiac decompensation, or when solitary cardiac disease exists with a controlled primary tumor and good prognosis. In cases of tamponade, urgent pericardiocentesis or a pericardial window can be life-saving. Arrhythmias due to myocardial infiltration may require medications or interventional procedures. The main goals are symptom relief and quality of life improvement, as curative treatment for cardiac metastasis is generally not feasible. Palliative care is the typical approach.

In light of the above, the involvement of a multidisciplinary team is crucial in the evaluation and management of patients with CMs so that the most appropriate therapeutic approach may be tailored [41,42,43,44,45,46,47,48,49,50,51,52,53,54,55,56,57,58,59,60,61,62,63,64,65] (Figure 7).

## 12. Oncologic Significance and Outcome of Cardiac Metastasis

Although rare, CM signifies widespread systemic disease and is typically associated with a poor prognosis, often representing the terminal stages of cancer. CMs are frequently identified postmortem, as they tend to be asymptomatic or present with non-specific symptoms. However, once symptoms occur, they are generally severe and can significantly impair the patient’s quality of life and survival. The prognosis for cardiac metastasis is poor, with survival typically measured in months following diagnosis, particularly in the presence of significant hemodynamic compromise. Treatment is primarily palliative, focusing on symptom management—such as controlling effusions or arrhythmias—rather than directly targeting the cardiac metastasis. Survival is influenced by multiple factors, including the stage and type of the primary malignancy, the presence of additional metastases (e.g., to the liver or brain), the site of cardiac involvement (pericardial, myocardial, or endocardial), and the efficacy of palliative measures, such as the management of pericardial effusions.

In cases of pericardial metastasis, fluid accumulation between the heart and the pericardium can lead to pericardial effusion, which may progress to cardiac tamponade, a condition with profound hemodynamic consequences. Myocardial infiltration can result in atrial or ventricular arrhythmias, heart block, or heart failure due to impaired contractile function. Endocardial metastasis may lead to valvular dysfunction, which increases the risk of heart failure and embolism. Additionally, superior vena cava syndrome or venous obstruction can impair venous return, causing venous hypertension, edema, and a reduction in cardiac output, potentially leading to systemic congestion and multi-organ failure.

## 13. Future Directions

CMs, although relatively rare, present significant challenges in clinical practice due to their late detection and complex pathophysiology.

Future research in this field is increasingly focused on developing targeted therapeutic interventions and improving diagnostic accuracy through multimodal imaging.

**Figure 8:**
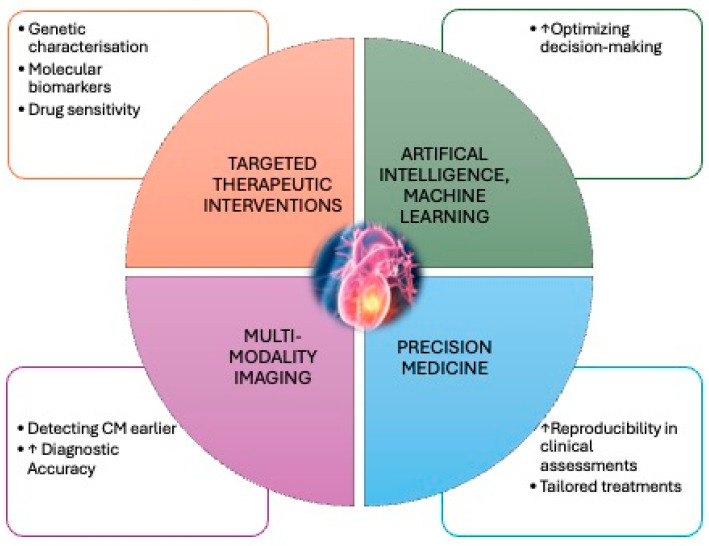
Future research in cardiac metastasis.

On the therapeutic front, there is a growing interest in precision medicine [66], utilizing genetic characterization and molecular biomarkers to tailor treatments specific to the metastatic tumor’s origin and its interaction with the cardiac microenvironment (Figure 8).

The evolution of precision medicine in oncology transforms our knowledge of cancer biology, enabling a more comprehensive approach to managing metastatic disease, including CMs. This paradigm shift involves integrating multiple biological dimensions to model the mechanisms underlying cancer progression in individual patients. The challenge arises from discovering multiple tumor drivers, particularly in metastatic sites such as the heart, which complicates clinical decision-making and broadens the range of therapeutic options [66].

Drug sensitivity in CMs depends on the actionability of specific targets, their clonal or subclonal origins, and their interplay with coexisting genomic and epigenomic alterations. Sequencing technologies have unveiled diverse genetic drivers that frequently emerge during disease progression or treatment resistance. These insights provide opportunities to identify novel therapeutic targets, inform clinical trials, and explore drug repurposing strategies.

It is critical to rank genomic alterations based on their established relevance and clinical actionability to optimize therapeutic prioritization in cardiac metastases. Furthermore, addressing the spatial and temporal heterogeneity of metastatic lesions, particularly within the complex cardiac microenvironment, is paramount. The advent of high-throughput technologies and the increasing availability of large-scale biological data necessitate the implementation of artificial intelligence (AI) algorithms for deeper analyses and actionable insights. AI, mainly its subset Machine Learning (ML), offers transformative potential in oncology by analyzing complex datasets, interpreting intricate biological information, and optimizing decision-making in specific clinical tasks. AI applications span the entire continuum of care for patients with CMs, from early detection and diagnosis to treatment planning and long-term management.

AI-based models can enhance diagnostic accuracy by refining imaging interpretation and developing predictive biomarkers tailored to the unique characteristics of cardiac metastases. These tools aim to improve reproducibility and precision in clinical assessments, thereby enabling the earlier detection and more reliable evaluation of metastatic disease in the heart. Integrating AI-driven methodologies into precision oncology holds particular promise in identifying patient-specific therapeutic strategies, ensuring that treatment is more targeted and aligned with individual tumor biology. In the context of CMs, AI-guided algorithms can support the development of personalized treatment protocols by predicting therapeutic responses. Such models could stratify patients based on their likelihood of responding to standard therapies, enabling the escalation of treatment intensity for resistant cases while de-escalating it for those predicted to respond effectively. This approach optimizes therapeutic outcomes and minimizes the risk of adverse events and treatment-related toxicities. Although many AI-based models for managing CMs remain experimental, ongoing advancements suggest their potential integration into clinical practice soon. By supporting physicians in complex decision-making processes, AI can change the management of cardiac metastases, ultimately improving patient outcomes and advancing the field of precision medicine.

Targeted therapies, such as immune checkpoint inhibitors and monoclonal antibodies, hold promise in offering more effective treatment options with fewer side effects compared to traditional chemotherapy. Additionally, advancements in multimodal imaging techniques, including positron emission tomography (PET), CMRI, and contrast-enhanced ultrasound, enhance the ability to detect CMs earlier and more accurately. When combined with AI and MC algorithms, these imaging methods can assist in identifying subtle changes in cardiac morphology and function, enabling the more precise monitoring of disease progression and therapeutic response. Ultimately, integrating personalized therapeutic approaches with advanced imaging modalities can significantly improve outcomes for patients with cardiac metastases, facilitating earlier detection, more effective treatments, and enhanced monitoring of therapeutic efficacy.

## 14. Conclusions

Although cardiac metastasis is uncommon, it is associated with significant morbidity and mortality due to its detrimental effects on cardiac function. Depending on the location and extent of metastatic involvement, it can result in severe hemodynamic instability, arrhythmias, heart failure, and cardiac tamponade. Management is predominantly palliative, aiming to alleviate symptoms and improve the quality of life of patients with advanced metastatic disease.

Imaging plays a critical role in the assessment of suspected cardiac tumors. With the increasing availability of multi-modality imaging, the ability to differentiate cardiac masses has improved, thereby facilitating optimal medical management. Imaging not only helps to identify potential causes of symptoms but also guides further diagnostic investigations and informs treatment strategies.

## Figures and Tables

**Table 1:** Summary of studies addressing cardiac metastases.

Authors	N° of Pts	Average Age (Years)	Incidence	Type of Cancer	Site of CM	Mean Survival Time	Management
Lin et al. [2].	41	52.6	12%	Lymphoma Lung cancer Cervical cancer Renal cancer Melanoma Esophageal cancer Colorectal cancer Soft tissue sarcoma Liver cancer Bone sarcoma Uncertain pathologic type	Left atrium Left ventricle Right atrium Right ventricle Interventricular septum Pericardium Others	19.70 Months	13 patients: chemotherapy alone 11 patients: chemotherapy + immunotherapy, radiotherapy, or targeting drugs 2 patients: surgery + radiotherapy or targeting drugs 1 patient: targeting drugs alone 1 patient: radiotherapy alone
Liu L. [19]	412 total 42 CM	57	7.7%	Lung cancer Cervical cancer Renal cancer Melanoma Esophageal cancer Colorectal cancer Soft tissue sarcoma Liver cancer	Left atrium Left ventricle Right atrium Right ventricle Interventricular septum Pericardium Valve Others	38.21 months	Surgery + radiotherapy + chemotherapy
Guan T. [20]	218	65–71	32.1%	Lymphoma Sarcoma Mesotheliomia and others	-	-	Surgery 52.3%
Liu L. [21]	389 total 75 CM	59 ± 14	19%	-	Left atrium Left ventricle Right atrium Right ventricle Interventricular septum Pericardium Valve	<2 years	Radiotherapy or chemotherapy
Luiz M. Nova-Camacho [22]	1294 autopsies 61 CM		4.71%	Lung cancer Melanoma Esophageal cancer Breast cancer Gastrointestinal cancer Pancreatobiliar cancer Skin cancer Pleura cancer Endocrine cancer Head and neck cancer	Left atrium Left ventricle Right atrium Right ventricle Interventricular septum Pericardium Valve	2.76 ± 3.85 years	-
Lau C. [23]	180	55.9 ± 16.4	39.4%	Sarcomas Renal cell cancer lung cancer Mesothelioma Teratoma Melanoma Thymoma carcinoid		18.3 ± 4.4 months	Neoadjuvant therapy + surgery
Kopoic K. [24]	12,285 autopsies		10–20%	Pleural mesothelioma (48.4%) Adenocarcinoma or squamous cell carcinoma of lung Breast carcinoma (15.5%) Ovarian carcinoma (10.3%) Bronchoalveolar carcinomas Lymphomyeloproliferative neoplasms (9.4%) Gastric carcinoma (8%) Renal carcinoma Pancreatic carcinoma Melanoma Esophageal cancer Colorectal carcinoma Leukemia Neuroendocrinal cancer Testicular germ cell cancer	Left atrium Left ventricle Right atrium Right ventricle Interventricular septum Pericardium		Focus on managing primary tumor Perioperative chemotherapy Surgery (including pericardiocentesis or pericardial window for effusion)

**Table 2:** Incidence of cardiac metastasis based on primary type of cancer.

Primary Cancer Type	Incidence	Features
Lung Cancer	~36–39%	Most common cancer type to metastasize to the heart.
Breast Cancer	~10–12%	Second-most common source of cardiac metastasis.
Melanoma	~28–64%	High incidence due to the aggressive nature of melanoma in spreading to various organs, including the heart.
Renal Cell Carcinoma	~7–20%	Notably metastatic to both cardiac and vascular structures.
Lymphomas and Leukemias	~9–24%	Includes non-Hodgkin lymphoma and other hematologic cancers which can spread to the pericardium, myocardium, or endocardium.
Gastrointestinal Cancers	~3–6%	Less commonly metastasizes to the heart, but possible in advanced disease stages.
Sarcomas	~10–15%	Soft-tissue sarcomas, especially angiosarcomas, may directly invade or metastasize to the heart.
Head and Neck Cancers	~1–3%	Rare incidence of cardiac metastasis; typically in advanced stages of metastatic disease.
Thyroid Cancer	<1%	Rarely metastasizes to the heart; typically involves the pericardium when it does.
Hepatocellular Carcinoma	~1–5%	Low incidence; metastasis to the heart occurs mainly in cases of vascular invasion.

**Table 3:** A comparative overview of the differential diagnosis between cardiac metastases and other cardiac masses.

	Cardiac Metastasis	Other Cardiac Masses
	Secondary to cancer elsewhere (e.g., lung, breast, melanoma)	Primary tumors (e.g., myxomas, fibromas, sarcomas)
Common Locations	Multiple lesions, often involving pericardium, myocardium	Specific sites: left atrium (myxoma), myocardium (fibroma), valves (papillary fibroelastoma)
Characteristics	Variable; can be multiple, diffuse, or infiltrative	Well-defined, specific to type (e.g., pedunculated, solid, infiltrative)
Imaging Features	Often multiple lesions, diffuse involvement; may show systemic cancer signs	Typically well-defined masses; can cause obstruction or local effects
Symptoms	Symptoms related to primary cancer, heart failure, arrhythmias	Symptoms related to mass effect, obstruction, or arrhythmias
Histology	Identifies cancer cells from the primary malignancy	Specific to tumor type (e.g., myxoma, fibroma, sarcoma)
Primary Cancer History	Often present or suspected	Usually absent or less relevant
Clinical Context	Evidence of systemic cancer, other metastatic sites	Usually isolated to the heart or part of a syndrome
Diagnostic Tools	Imaging (CT, MRI, PET) and biopsy; look for systemic malignancy	Imaging (echocardiography, MRI, CT) and biopsy if needed
